# Sellar germinoma mimicking IgG4-related hypophysitis: a case report

**DOI:** 10.1186/s12902-021-00930-3

**Published:** 2022-01-15

**Authors:** Kang Chen, Yong Yao, Xinxin Mao, Hui You, Linjie Wang, Lian Duan, Kan Deng, Wen Zhang, Xin Lian, Huijuan Zhu

**Affiliations:** 1 Department of Endocrinology, Key Laboratory of Endocrinology of National Health Commission, Translation Medicine Centre, Peking Union Medical College Hospital, Peking Union Medical College, Chinese Academy of Medical Sciences, No.1 Shuaifuyuan, Wangfujing Street, Dongcheng District, Beijing, 100730 China; 2 Department of Neurosurgery, Peking Union Medical College Hospital, Peking Union Medical College, Chinese Academy of Medical Sciences, Beijing, China; 3 Department of Pathology, Peking Union Medical College Hospital, Peking Union Medical College, Chinese Academy of Medical Sciences, Beijing, China; 4 Department of Radiology, Peking Union Medical College Hospital, Peking Union Medical College, Chinese Academy of Medical Sciences, Beijing, China; 5 Department of Rheumatology, Peking Union Medical College Hospital, Peking Union Medical College, Chinese Academy of Medical Sciences, Beijing, China; 6 Department of Radiation Oncology, Peking Union Medical College Hospital, Peking Union Medical College, Chinese Academy of Medical Sciences, Beijing, China

**Keywords:** IgG4-related disease, Hypophysitis, Germinoma

## Abstract

**Background:**

The differential diagnosis of IgG4-related hypophysitis and other inflammatory diseases or tumors involving sellar region is challenging even after sellar biopsy. Sellar germinoma is usually infiltrated by lymphocytes or plasma cells, and may be confused with hypophysitis.

**Case presentation:**

A 36-year-old man with diabetes insipidus, elevated serum IgG4 level (336 mg/dl), and sellar mass was suspected to have IgG4-related hypophysitis, and no other lesion of IgG4-related disease was detected. After treated by prednisone and mycophenolate mofetil, the serum IgG4 decreased to 214 mg/dl. However, after withdrawal of the drugs, the IgG4 level increased to 308 mg/dl. Endocrine assessments revealed panhypopituitarism, and the sellar mass enlarged. Transsphenoidal sellar exploration and biopsy was conducted. Pathological examination showed that the lesion was germinoma with lymphocytes and plasma cells infiltration, and IgG4-staining was positive (70/HPF, IgG4/IgG ratio = 10%). The patient was then treated by cisplatin and etoposide. After four cycles of chemotherapy, the serum IgG4 was 201 mg/dl, and the sellar mass was invisible.

**Conclusion:**

Sellar germinoma can mimic the clinical characteristics of IgG4-related hypophysitis. Poor response to glucocorticoids can be used as an exclusion criterion in the clinical diagnosis of IgG4-related hypophysitis.

## Background

IgG4-related disease is a spectrum of immune-mediated conditions characterized by tumefactive lesions infiltrated by IgG4-producing plasma cells or lymphocytes [1, 2]. It could affect one or more organs such as the pancreas, biliary ducts, salivary glands, and orbits, while pituitary gland involvement is less common [3]. IgG4-related hypophysitis was increasingly recognized after the report of the first case in 2004 [4], and the first pathologically confirmed case in 2007 [5]. While pooled data showed that IgG4-related hypophysitis only accounted for 1.3–4% of primary hypophysitis [6, 7], its prevalence was up to 30–40% in some centers [8, 9]. Similar to other types of hypophysitis, IgG4-related hypophysitis manifests as anterior pituitary hormone deficiency and/or central diabetes insipidus, as well as symptoms caused by mass effects of the lesion [10, 11]. Moreover, serum IgG4 level was increased in many patients, and the involvement of other organs was common [6]. IgG4-related hypophysitis might be easily confused with other diseases involving the pituitary, including benign or malignant tumors, but the former one could be treated by glucocorticoids while the latter ones might need surgery, chemotherapy or radiotherapy [11]. Pathological examination played a critical role in the current diagnostic criteria of IgG4-related hypophysitis [10, 11], but sometimes its differential diagnosis might remain challenging even after biopsy. For example, it was reported that pituitary involvement of granulomatosis with polyangiitis could not only mimic the symptoms of IgG4-related hypophysitis but also share some pathological features [12], and some other types of pituitary inflammatory lesions could also be infiltrated by IgG4-positive cells [13].

Intracranial germ-cell tumors (GCTs) mainly affect children and adolescents, while adult patients are relatively rare [14]. The lesion typically locates at the pineal and suprasellar region, and sometimes inside the sella turcica [15]. Similar to other diseases that involved the hypothalamus and pituitary, suprasellar GCT can manifest as deficiencies in pituitary hormones and symptoms related to local compression [16, 17]. The most common type of intracranial GCTs is germinoma, which accounts for about 70% of the cases [12, 17]. Germinoma is so immunogenic that the tumor tissue could be infiltrated by numerous lymphocytes or plasma cells, which makes it difficult to be differentiated from hypophysitis on some occasions [10, 18, 19].

We report here a patient presented with diabetes insipidus and hypopituitarism who was initially misdiagnosed as IgG4-related hypophysitis. This patient fulfilled the clinical diagnostic criteria of IgG4-related hypophysitis, but consequent biopsy of the pituitary lesion revealed that it was actually an intracranial germinoma.

## Case presentation

**Fig. 1:**
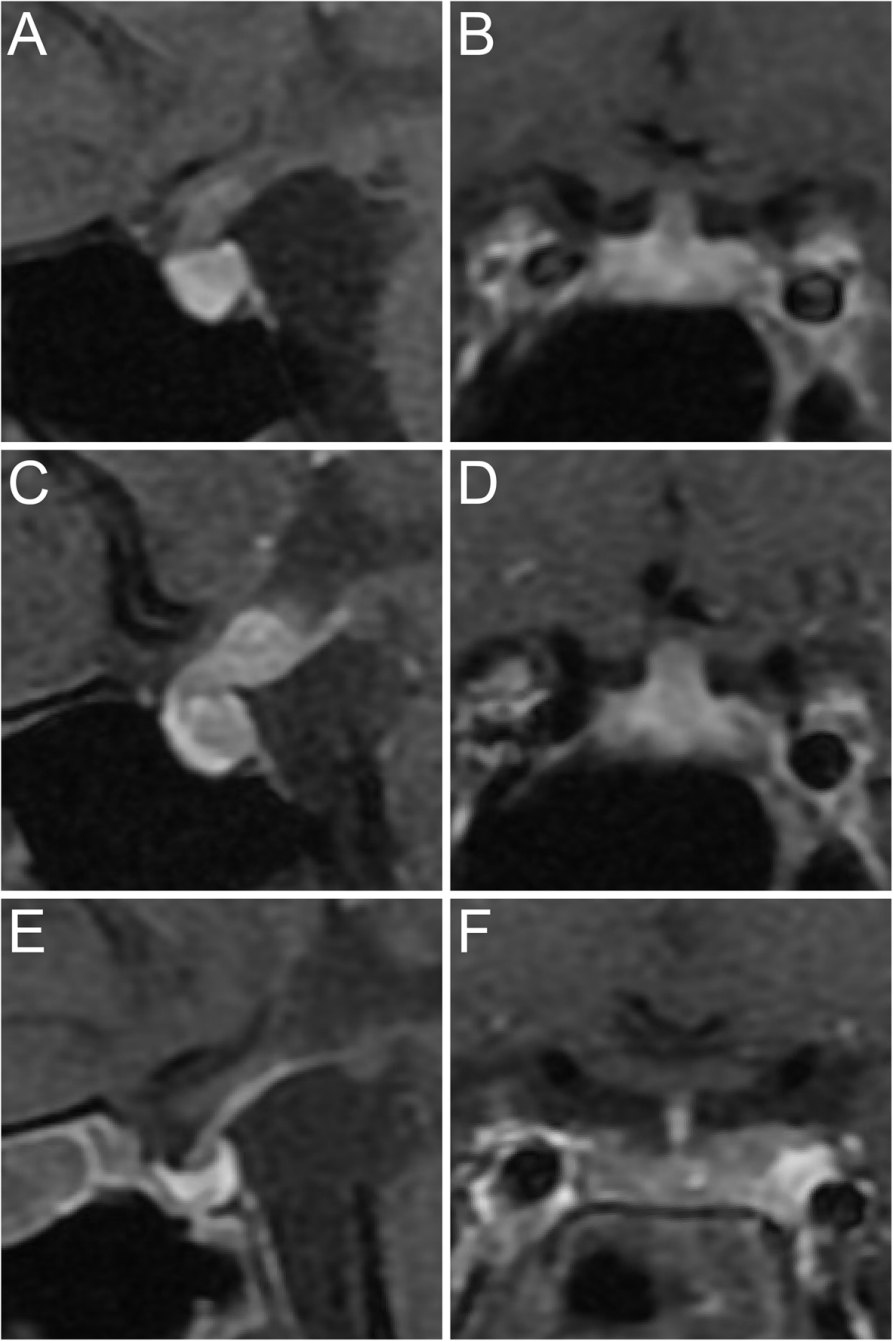
Contrast enhanced MRI of the pituitary. (**A, B**) Sellar mass and pituitary stalk thickening before treatment. (**C, D**) The sellar and stalk lesion progressed after treatment by prednisone and mycophenolate mofetil. (**E, F**) The sellar and stalk lesion shrank after four cycles of chemotherapy

In April 2019, a 36-year-old man was referred to our hospital because of polyuria and polydipsia for about 9 months. His urine volume reduced from 10 L/day to 5 L/day after oral administration of desmopressin (0.05 mg b.i.d.) for two weeks, but the serum osmolarity was still 317 mOsm/kgH_2_O (normal range: 275–305 mOsm/kgH_2_O) and serum Na^+^ was 145 mmol/L (normal range: 135-145 mmol/l). Blood glucose was normal and urine glucose was negative. Endocrine assessment of anterior pituitary was generally normal [TSH 2.258 μIU/mL (normal range: 0.380–4.340), FT4 1.23 ng/dl (normal range: 0.81–1.89 ng/dl), FT3 3.99 pg/ml (normal range: 1.80–4.10 pg/ml); ACTH 59.0 pg/ml (normal range: 0–46 pg/ml), cortisol 23.1 μg/dl (normal range: 4.0–22.3 μg/dl); LH 2.61 IU/L (normal range: 1.24–8.62 IU/L), FSH 4.00 IU/L (normal range: 1.27–19.26 IU/L), T 2.97 ng/ml (normal range: 1.75–7.81 ng/ml); IGF1 133 ng/ml (normal range: 109–284 ng/ml); prolactin 9.1 ng/ml (normal range: 2.64–13.13 ng/ml)]. Although the cortisol was slightly higher, the patient had no symptom or sign of hypercortisolism. The serum IgG4 (336 mg/dl) (normal range: 8–140 mg/dl) and IgE (399 IU/ml) (normal range: 0–60 IU/ml) concentration was elevated. Contrast enhanced MRI demonstrated a mass occupying posterior pituitary and pituitary stalk, and the diameter of the pituitary stalk was 3.4 × 3.4 mm (Fig. 1A, B). Chest and abdominal CT did not detect other lesion except for liver steatosis and cholelithiasis. Based on these findings, a diagnosis of IgG4-related hypophysitis was suspected.

**Fig. 2:**
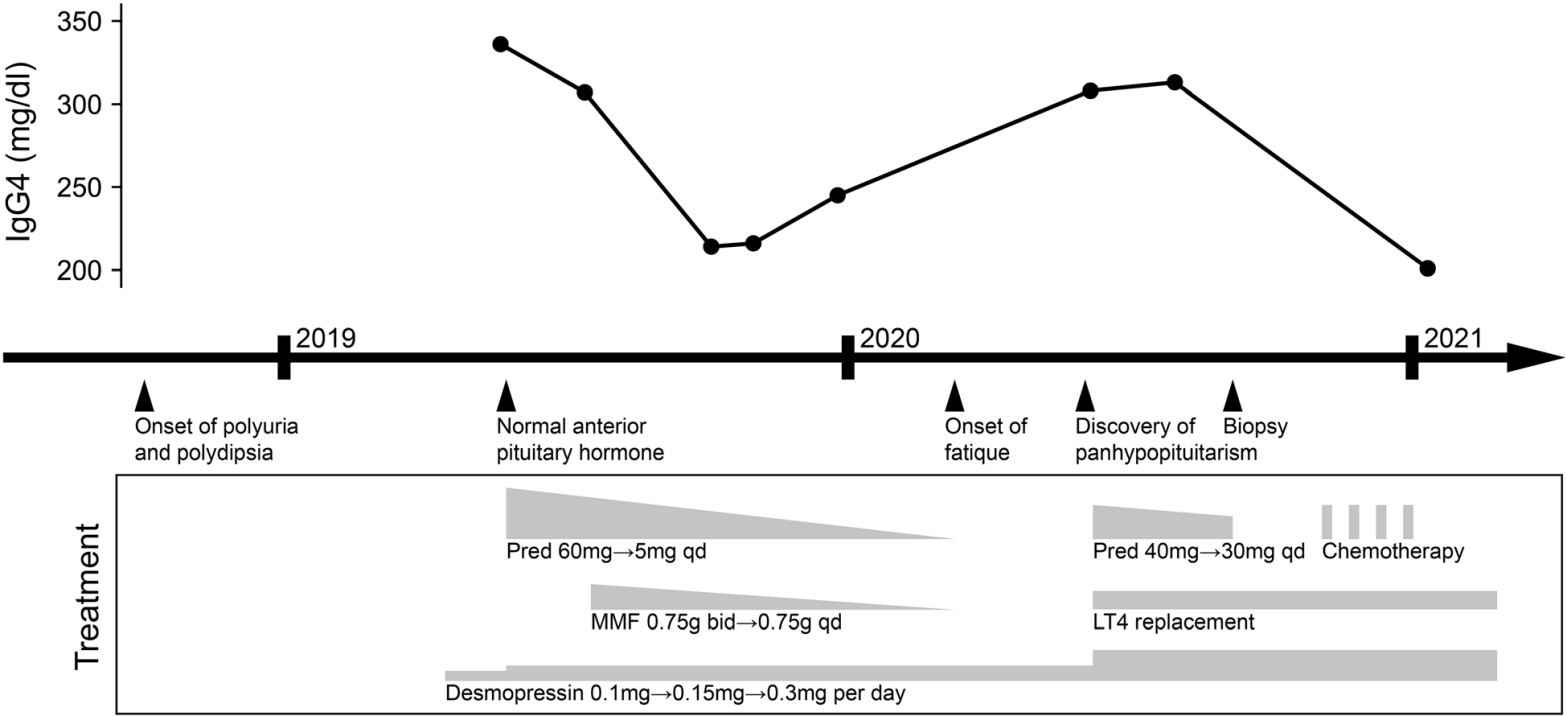
Timeline of treatment and changes of serum IgG4 level. Pred: prednisone, MMF: mycophenolate mofetil, LT4: levothyroxine

Prednisone treatment was initiated at a dose of 60 mg q.d. in April 2019. The dose of prednisone was tapered to 40 mg q.d. in June 2019, and mycophenolate mofetil of 0.75 mg b.i.d. was added. The serum IgG4 level gradually decreased and reached its nadir (214 mg/dl) in September 2019 (Fig. 2), but desmopressin (0.05 mg t.i.d.) was still needed to control the symptoms of diabetes insipidus. In December 2019, the dose of mycophenolate mofetil was decrease to 0.75 mg q.d., and prednisone was also tapered gradually. In February 2020, prednisone was finally tapered to 5 mg q.d.. The patient stopped taking the drugs by himself, and developed fatigue after that. In June 2020, the serum IgG4 increased to 308 mg/dl. Endocrine assessments revealed panhypopituitarism (TSH 0.071μIU/mL, FT4 0.50 ng/dl, FT3 3.50 pg/ml; ACTH 7.4 pg/ml, cortisol < 0.50 μg/dl; LH < 0.2 IU/L, FSH 1.70 IU/L, T < 0.1 ng/ml; IGF1 89 ng/ml), and prolactin level elevated to 32.6 ng/ml. Pituitary MRI showed that the lesion enlarged and involved the hypothalamus, and the diameter of the pituitary stalk was 5.1 × 4.7 mm (Fig. 1C, D). Replacement therapy with levothyroxine was given and prednisone treatment was restarted at a dose of 40 mg q.d.. Here the dose of prednisone was lower than the initial dose of the first course, since the patient had adverse effects including weight gain and hypertriglyceridemia. However, the IgG4 level did not drop, and the diameter of the pituitary stalk further enlarged to 6.0 × 8.6 mm (figure not shown).

**Fig. 3:**
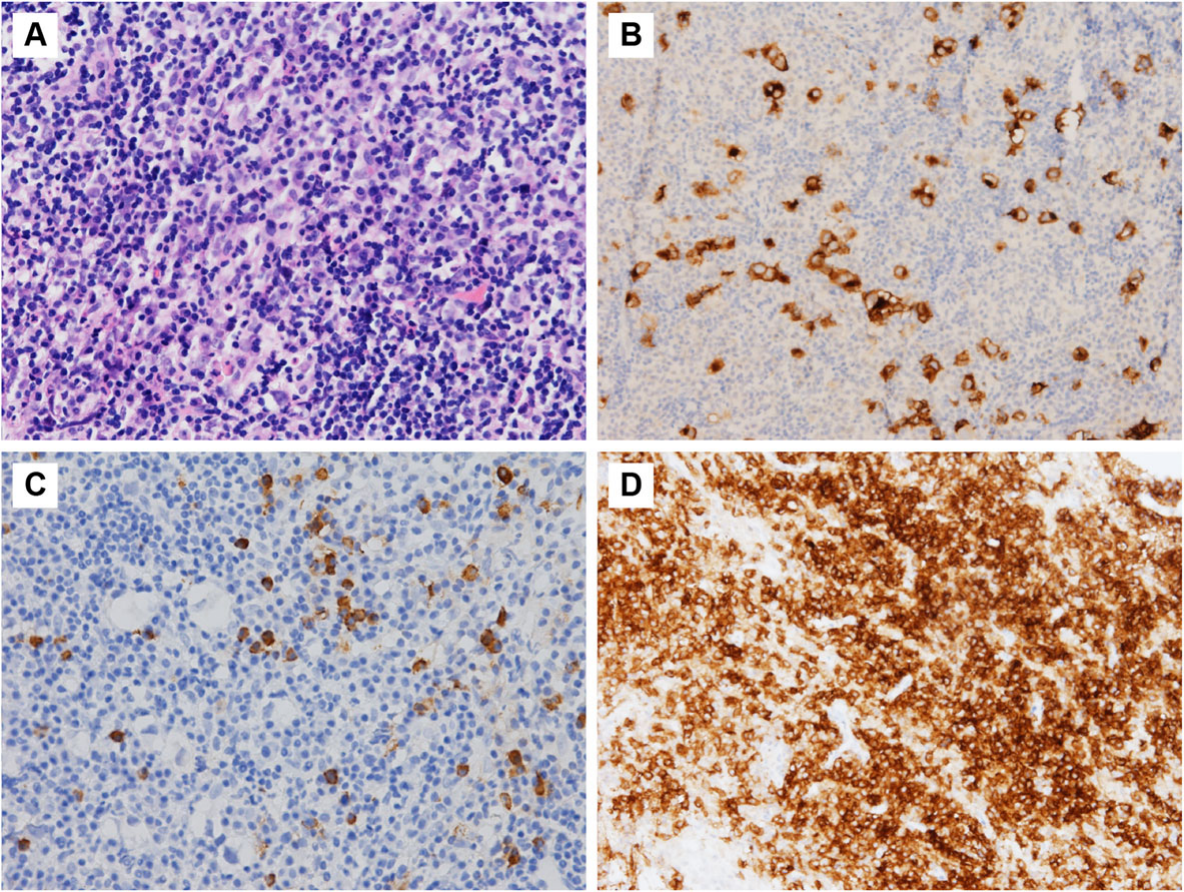
Histopathology of the pituitary lesion. **A** HE staining shows scattered large tumor cells with round nuclei and prominent nucleoli, and dense lymphoplasmacytic infiltration. **B** Immunohistochemistry staining for CD117 (a marker of germinoma) is positive in tumor cells. **C** Immunohistochemistry staining for IgG4. **D** Immunohistochemistry staining for CD38 (a marker of plasma cells)

The patient was admitted to our hospital for further evaluation. Physical examination found no sign of lacrimal gland or salivary gland swelling. Daily urine volume was 1500-2000 ml under desmopressin of 0.1 mg t.i.d., and increased to 2800 ml when desmopressin was suspended for one day. In the cerebrospinal fluid, the βhCG and AFP were not elevated [βhCG 2.96 IU/L (normal range: < 5 IU/L), AFP undetectable], and no tumor cell was found. The thyroid ultrasonography and abdominal CT results were generally normal, and 18F-FDG-PET showed modest FDG uptake at the pituitary stalk (SUVmax = 1.0). Neurosurgeons conducted transsphenoidal sellar exploration and biopsy for the patient in August 2020, and found that the grayish-white lesion compressed the pituitary and invaded the suprasellar region through the pituitary stalk. Pathological examination (Fig. 3) confirmed that the lesion was germinoma with positive CD117 and OCT4 immunohistochemical staining. The tumor was infiltrated by mononuclear cells including T cells (CD3+), B cells (CD20+), and plasma cells (CD38+/CD138+). IgG4 staining was positive (70/HPF), and the IgG4/IgG ratio was 10%.

Four cycles of chemotherapy consisting of cisplatin and etoposide were given from Oct 2020 to Dec 2020. The sellar and suprasellar mass was invisible on the pituitary MRI evaluated after two and four cycles of chemotherapy, and the diameter of the pituitary stalk was 2.2 × 2.1 mm (Fig. 1E, F). The serum IgG4 level dropped to 201 mg/dl one month after the last cycle of chemotherapy.

## Discussion and conclusion

In this case report we described a patient with sellar germinoma who was misdiagnosed as IgG4-related hypophysitis, which is the first case of sellar germinoma with elevated IgG4 level. This case reminds us that germinoma may be a mimicker of IgG4-related hypophysitis, and the diagnosis of IgG4-related hypophysitis should be made after thorough assessments to exclude other diseases.

The classification criteria of IgG4-RD have been published by the American College of Rheumatology/European League Against Rheumatism (ACR/EULAR) in 2019 [2], but patients with isolated pituitary involvement were excluded in the development of these criteria due to its relatively low prevalence. Thus, the criteria for IgG4-related hypophysitis need to be specially developed. The first set of diagnostic criteria for IgG4-related hypophysitis (Table 1) was proposed by Leporati et al. in 2011 based on the features of 12 patients, in which the pathological finding alone was enough to establish the diagnosis of IgG4-related hypophysitis. When the pituitary biopsy was not available, the diagnosis could also be made according to the image finding, serum IgG4 level, pathology of other involved organs, or the response to glucocorticoids [10]. Another set of criteria (Table 1) was proposed by the Japan Endocrine Society in 2020, in which the diagnosis was classified as definitive, probable, or possible one. Pathology of biopsy was essential to establish the definitive diagnosis, while the clinical diagnosis with different certainty could also be made according to the symptoms, laboratory examinations, and image studies [11].

**Table 1:** The diagnostic criteria of IgG4-related hypophysitis

	2011 Laporati [10]^a^			2020 Japan [11]					
				Definitive		Probable		Possible	
Symptoms due to mass effects, hypopituitarism, or central diabetes insipidus				√	√	√	√	√	√
Laboratory findings of hypopituitarism				√		√		√	
Decreased response in stimulation tests				√		√		√	
Laboratory findings of central diabetes insipidus					√		√		√
Image findings: pituitary enlargement/mass, stalk thickening		√	√	√	√	√	√	√	√
Elevated serum IgG4			√					√	√
Pathology of pituitary	√			√	√				
Pathology of other involved organs		√				√	√		
Response to glucocorticoids			√						

a. IgG4-related hypophysitis can be diagnosed when all items in any of the columns are fulfilled

No matter which set of criteria was adopted, the symptoms of CDI, laboratory examinations of hypopituitarism, pituitary mass and stalk thickening on MRI, and elevated serum IgG4 were all in accordance with the clinical diagnosis of IgG4-related hypophysitis in the current case. However, some features of this patient were distinct from typical IgG4-related hypophysitis. In previous reports, male patients with IgG4-related hypophysitis were usually elderly [6, 20], while this patient is relatively young. Moreover, the symptoms did not significantly relieve and the serum IgG4 did not drop to normal range after prednisone and MMF treatment. Meanwhile, panhypopituitarism emerged and MRI revealed the progression of the sellar lesion. In fact, if the image follow-up had been done during the initial stage of the treatment, such progression might have been detected earlier. These made the clinical diagnosis of IgG4-related hypophysitis in this case questionable, and indicated that the diagnostic criteria of IgG4-related hypophysitis might need some modifications.

The 2019 ACR/EULAR classification criteria for IgG4-RD consists of entry criteria, exclusion criteria, and a scoring system. Similarly, we believed that adding exclusion criteria to the diagnostic criteria of IgG4-related hypophysitis would be helpful, though it may be too rare to develop such a scoring system. IgG4-related hypophysitis is a benign disease that can be treated by glucocorticoid [10], so other diseases that need more aggressive therapies should be ruled out before the establishment of this diagnosis. In a review of previous cases, all the IgG4-related hypophysitis patients responded to glucocorticoids except for two patients who received a low dose of hydrocortisone [6]. We suggested that poor response to glucocorticoids should be adopted as an exclusion criterion in the clinical diagnosis of IgG4-related hypophysitis, since it is indicative of malignancies.

The pathological diagnosis of IgG4-RD relies on both morphological findings and immunohistochemical staining for IgG4. The key morphological features include dense lymphoplasmacytic infiltration, storiform fibrosis, and obliterative phlebitis, but variable features may be present in some organs [21]. In IgG4-related hypophysitis, lymphoplasmacytic infiltration was seen in almost all the cases, while storiform fibrosis was less common, and obliterative phlebitis has not been reported yet [9, 20]. Thus, pathology criteria should be specifically modified for IgG4-related hypophysitis. As for the IgG4-positive cells, the criteria in the two sets of criteria were slightly different. In the Japan Endocrine Society criteria, pathological findings are in accordance with IgG4-related hypophysitis if either the density of IgG4-positive cells is > 10 per high-power field (HPF) or the ratio of IgG4/IgG-positive cells is > 40% [11], while only the density is considered in the Leporati criteria [10]. The current case shared some pathological features with IgG4-related hypophysitis, that is, dense lymphoplasmacytic infiltration and positive IgG4 staining. It should not be diagnosed as IgG4-related hypophysitis due to the presence of germinoma, but this might explain the elevated serum IgG4 in this case.

The relation between germinoma and the IgG4 reaction is unknown in this case. However, it could be speculated that the IgG4 reaction was secondary to germinoma, since no other organ was found to be involved by IgG4-RD in this patient, and serum IgG4 level dropped after the remission of germinoma. IgG4-RD was generally considered to be initiated by autoimmunity, followed by activation of type 2 helper T cells and regulatory T (Treg) cells, which can prompt the production of IgG4 [1]. Previous studies reported the presentation of anti-pituitary antibodies in the serum of patients with IgG4-related hypophysitis, further supporting this hypothesis [22, 23]. Tumors could also trigger a similar process, and it was reported that tumor cells per se could play the role of antigen-presenting cells or Treg, which might explain the elevation of serum IgG4 and peritumoral infiltration of IgG4-positive cells in cancers such as pancreatic cancer or cholangiocarcinoma [24–26]. In other types of secondary hypophysitis caused by pituitary adenoma, Rathke cyst or craniopharyngioma, infiltration of IgG4-positive cells was also observed [13]. The exposure of autoantigens may also present in germinoma, which was hypothesized to be the reason for florid lymphocyte infiltration in part of germinoma cases [18, 27]. However, no previous case of IgG4 reaction in germinoma was reported, and the current case may be the first such case. On the other hand, IgG4 may be involved in carcinogenesis through immune evasion, and higher IgG4 level and more IgG4-positive lymphocytes infiltration were associated with worse outcomes [28]. In the current case, the sellar germinoma responded well to routine therapy despite the IgG4 reaction. Further studies are needed to clarify whether IgG4 reaction can influence the clinical characteristics and treatment outcome of sellar germinoma.

In summary, we present the first case of sellar germinoma mimicking the clinical and pathological features of IgG4-related hypophysitis. The diagnostic criteria of IgG4-related hypophysitis may need some modifications, and the response to glucocorticoid should be taken into consideration. Besides, it provided clues to understand the role of IgG4 in the pathogenesis of primary or secondary hypophysitis.

## Data Availability

Data sharing is not applicable to this article as no datasets were generated or analyzed during the current study.

## Abbreviations

IgG4: immunoglobulin G4
GCT: germ-cell tumor
MRI: magnetic resonance imaging
TSH: thyroid stimulating hormone
FT4: free thyroxine
FT3: free triiodothyronine
ACTH: adrenocorticotropic hormone
IGF1: insulin-like growth factor 1
Pred: prednisone
MMF: mycophenolate mofetil
LT4: levothyroxine
βhCG: beta human chorionic gonadotropin
AFP: alpha fetoprotein
18F-FDG: 18F-fluorodeoxyglucose
PET-CT: positron emission tomography-computerized tomography
SUV: standardized uptake value
HPF: high-power field
IgG4-RD: IgG4 related disease
CDI: central diabetes insipidus
Treg: regulatory T cell

## References

[1] Stone JH, Zen Y, Deshpande V (2012). IgG4-related disease. N Engl J Med.

[2] Wallace ZS, Naden RP, Chari S, Choi HK, Della-Torre E, Dicaire JF, Hart PA, Inoue D, Kawano M, Khosroshahi A, Lanzillotta M, Okazaki K, Perugino CA, Sharma A, Saeki T, Schleinitz N, Takahashi N, Umehara H, Zen Y, Stone JH, Members of the ACR/EULAR IgG4-RD Classification Criteria Working Group (2020). The 2019 American College of Rheumatology/European league against rheumatism classification criteria for IgG4-related disease. Ann Rheum Dis.

[3] Kamisawa T, Zen Y, Pillai S, Stone JH (2015). IgG4-related disease. Lancet (London, England).

[4] van der Vliet HJ, Perenboom RM (2004). Multiple pseudotumors in IgG4-associated multifocal systemic fibrosis. Ann Intern Med.

[5] Wong S, Lam WY, Wong WK, Lee KC (2007). Hypophysitis presented as inflammatory pseudotumor in immunoglobulin G4-related systemic disease. Hum Pathol.

[6] Shikuma J, Kan K, Ito R, Hara K, Sakai H, Miwa T, Kanazawa A, Odawara M (2017). Critical review of IgG4-related hypophysitis. Pituitary..

[7] Caturegli P, Di Dalmazi G, Lombardi M, Grosso F, Larman HB, Larman T (2016). Hypophysitis secondary to cytotoxic T-lymphocyte-associated protein 4 blockade: insights into pathogenesis from an autopsy series. Am J Pathol.

[8] Bando H, Iguchi G, Fukuoka H, Taniguchi M, Yamamoto M, Matsumoto R, Suda K, Nishizawa H, Takahashi M, Kohmura E, Takahashi Y (2014). The prevalence of IgG4-related hypophysitis in 170 consecutive patients with hypopituitarism and/or central diabetes insipidus and review of the literature. Eur J Endocrinol.

[9] Bernreuther C, Illies C, Flitsch J, Buchfelder M, Buslei R, Glatzel M (2017). IgG4-related hypophysitis is highly prevalent among cases of histologically confirmed hypophysitis. Brain Pathol (Zurich, Switzerland).

[10] Leporati P, Landek-Salgado MA, Lupi I, Chiovato L, Caturegli P (2011). IgG4-related hypophysitis: a new addition to the hypophysitis spectrum. J Clin Endocrinol Metab.

[11] Takagi H, Iwama S, Sugimura Y, Takahashi Y, Oki Y, Akamizu T, Arima H (2020). Diagnosis and treatment of autoimmune and IgG4-related hypophysitis: clinical guidelines of the Japan Endocrine Society. Endocr J.

[12] Bando H, Iguchi G, Fukuoka H, Taniguchi M, Kawano S, Saitoh M, Yoshida K, Matsumoto R, Suda K, Nishizawa H, Takahashi M, Morinobu A, Kohmura E, Ogawa W, Takahashi Y (2015). A diagnostic pitfall in IgG4-related hypophysitis: infiltration of IgG4-positive cells in the pituitary of granulomatosis with polyangiitis. Pituitary..

[13] Nishioka H, Shibuya M, Haraoka J (2010). Immunohistochemical study for IgG4-positive plasmacytes in pituitary inflammatory lesions. Endocr Pathol.

[14] Committee of Brain Tumor Registry of Japan. Report of Brain Tumor Registry of Japan (1969–1996). Neurologia medico-chirurgica. 2003;43 Suppl:i-vii, 1–111. 10.2176/nmc.43.S1. 14705327

[15] Guzzo MF, Bueno CB, Amancio TT, Rosemberg S, Bueno C, Arioli EL (2013). An intrasellar germinoma with normal tumor marker concentrations mimicking primary lymphocytic hypophysitis. Arq Bras Endocrinol Metabol.

[16] Bromberg JE, Baumert BG, de Vos F, Gijtenbeek JM, Kurt E, Westermann AM (2013). Primary intracranial germ-cell tumors in adults: a practical review. J Neuro-Oncol.

[17] Kong Z, Wang Y, Dai C, Yao Y, Ma W, Wang Y (2018). Central nervous system germ cell tumors: a review of the literature. J Child Neurol.

[18] Pal R, Rai A, Vaiphei K, Gangadhar P, Dutaa P (2019). Intracranial Germinoma masquerading as secondary granulomatous Hypophysitis: a case report and review of literature. Neuroendocrinology..

[19] Louis DN, Ohgaki H, Wiestler OD, Cavenee WK (2007). WHO classification of Tumours of the central nervous system.

[20] Uccella S, Amaglio C, Brouland JP, Bianconi E, Ippolito S, Messerer M, Rouiller N, Tanda ML, Sessa F, la Rosa S (2019). Disease heterogeneity in IgG4-related hypophysitis: report of two histopathologically proven cases and review of the literature. Virchows Arch: Int J Pathol.

[21] Deshpande V, Zen Y, Chan JK, Yi EE, Sato Y, Yoshino T (2012). Consensus statement on the pathology of IgG4-related disease. Mod Pathol: Off J U S Can Acad Pathol Inc.

[22] Landek-Salgado MA, Leporati P, Lupi I, Geis A, Caturegli P (2012). Growth hormone and proopiomelanocortin are targeted by autoantibodies in a patient with biopsy-proven IgG4-related hypophysitis. Pituitary..

[23] Iwata N, Iwama S, Sugimura Y, Yasuda Y, Nakashima K, Takeuchi S, Hagiwara D, Ito Y, Suga H, Goto M, Banno R, Caturegli P, Koike T, Oshida Y, Arima H (2017). Anti-pituitary antibodies against corticotrophs in IgG4-related hypophysitis. Pituitary..

[24] Crescioli S, Correa I, Karagiannis P, Davies AM, Sutton BJ, Nestle FO, Karagiannis SN (2016). IgG4 characteristics and functions in Cancer immunity. Curr Allergy Asthma Rep.

[25] Harada K, Shimoda S, Kimura Y, Sato Y, Ikeda H, Igarashi S (2012). Significance of immunoglobulin G4 (IgG4)-positive cells in extrahepatic cholangiocarcinoma: molecular mechanism of IgG4 reaction in cancer tissue. Hepatology (Baltimore, Md).

[26] Brito-Zerón P, Bosch X, Ramos-Casals M, Stone JH (2016). IgG4-related disease: advances in the diagnosis and treatment. Best Pract Res Clin Rheumatol.

[27] Gutenberg A, Bell JJ, Lupi I, Tzou SC, Landek-Salgado MA, Kimura H, Su J, Karaviti LP, Salvatori R, Caturegli P (2011). Pituitary and systemic autoimmunity in a case of intrasellar germinoma. Pituitary..

[28] Wang H, Xu Q, Zhao C, Zhu Z, Zhu X, Zhou J (2020). An immune evasion mechanism with IgG4 playing an essential role in cancer and implication for immunotherapy. J Immunother Cancer.

